# Prokaryotic Microbial Diversity and Community Assembly in Reclaimed Coastal Agricultural Soils

**DOI:** 10.3390/microorganisms14010120

**Published:** 2026-01-06

**Authors:** Yifan Yin, Weidong Xu, Min Xu, Yuwei Wang, Hao Liu, Hui Cao, Feng Wang

**Affiliations:** 1 Key Laboratory of Agricultural Environmental Microbiology, Ministry of Agriculture and Rural Affairs, College of Life Sciences, Nanjing Agricultural University, Nanjing 210095, China; 2018216014@njau.edu.cn (Y.Y.); 2024216018@stu.njau.edu.cn (W.X.); 2023816085@stu.njau.edu.cn (M.X.); 2025216019@stu.njau.edu.cn (Y.W.); 2022216010@stu.njau.edu.cn (H.L.); 2 Institute of Eco-Environmental Sciences, Ningbo Academy of Agricultural Sciences, Ningbo 315040, China

**Keywords:** coastal reclamation, soil prokaryotes, community assembly, microbial biogeographical distribution, long-term chronosequence

## Abstract

Coastal reclamation profoundly alters soil physicochemical conditions and strongly influences soil microbial ecology; however, the millennial-scale successional patterns and assembly mechanisms of prokaryotic communities under such long-term disturbance remain insufficiently understood. In this study, we investigated archaeal and bacterial communities in the plow layer along a 0–1000-year coastal reclamation chronosequence on the southern shore of Hangzhou Bay. We analyzed community abundance, diversity, composition and assembly processes, and quantified the relative contributions of geographic distance, environmental factors and reclamation years to microbial biogeographic patterns. The results showed that reclamation markedly drove continuous soil desalination, acidification, nutrient accumulation, and particle-size refinement. Bacterial abundance exhibited a sharp decline during the early stages of reclamation, whereas archaeal abundance remained relatively stable. The α-diversity of both archaea and bacteria peaked at approximately 210–230 years of reclamation. Community assembly processes differed substantially between the two microbial domains: the archaeal communities were dominated by stochastic processes (77.78%) identified as undominated processes and dispersal limitation, whereas bacterial communities were primarily shaped by deterministic processes (70.75%) driven as variable selection. Distance–decay analysis indicated that bacterial communities were more sensitive to environmental gradients. Multiple regression and variance partitioning further demonstrated that soil pH and electrical conductivity were the key drivers of community structure. Overall, this study reveals the millennial-scale community dynamics and assembly mechanisms of archaea and bacteria in response to coastal reclamation, providing mechanistic insights into long-term microbial ecological succession and offering valuable guidance for sustainable agricultural management and ecological restoration in reclaimed coastal regions.

## 1. Introduction

Coastal wetlands are among the most productive and ecologically valuable ecosystems on Earth, playing critical roles in carbon sequestration, nutrient cycling, hydrological regulation, and the maintenance of biodiversity [1]. However, driven by population growth and increasing demand for agricultural land, large-scale reclamation of coastal wetlands has taken place in many regions over the past several centuries [2,3,4]. Converting coastal wetlands into arable land substantially alters their original physicochemical environment and initiates soil development processes that may last for hundreds to thousands of years [5,6,7,8]. Thus, understanding how reclamation reshapes soil properties and microbial ecological processes is essential for predicting microbial community succession and guiding sustainable land management.

Reclamation typically triggers a series of changes in soil physicochemical properties, which in turn lead to shifts in soil microbial communities. In coastal tidal flats, reclamation significantly decreases soil pH and salinity [9,10], both of which are key drivers of microbial community composition [11,12,13]. Previous studies have shown that soil electrical conductivity [14] and organic carbon [15] also undergo significant changes after reclamation, influencing microbial community dynamics. As primary drivers of carbon and nitrogen cycling and nutrient transformation, bacteria and archaea play essential roles during both reclamation and subsequent ecological restoration processes [16]. However, the two groups differ markedly in ecological strategies, environmental adaptability and metabolic potential. For instance, archaea often exhibit stronger adaptation to saline–alkaline environments, whereas bacteria are more responsive to nutrient conditions and physicochemical gradients [17]. Consequently, the environmental changes induced by reclamation are likely to exert differential impacts on these two microbial domains.

In recent years, microbial community assembly mechanisms have become a central topic in soil ecology. Ecological theory posits that microbial community structure is shaped by the interplay between deterministic processes that are driven by environmental filtering, species interactions, and niche differentiation, alongside stochastic processes such as drift, dispersal limitation, and historical contingencies [18]. Numerous studies have demonstrated that changes in soil physicochemical properties can substantially influence the relative contributions of deterministic versus stochastic forces. For example, under strong environmental stress or pronounced nutrient gradients, microbial communities often exhibit stronger environmental selection [19]. In contrast, along long-term successional trajectories, stochastic dispersal and ecological drift may become increasingly important as the environment stabilizes [20]. However, under the unique context of long-term anthropogenic disturbance from coastal land reclamation, questions remain unclear regarding how different microbial groups (e.g., archaea vs. bacteria) undergo community assembly dynamics over millennial time scales and how deterministic and stochastic processes evolve with reclamation years.

In this study, we investigated microbial responses along a coastal reclamation chronosequence spanning 0–1000 years, focusing on archaeal and bacterial communities in the plow layer. By integrating analysis of microbial abundance, diversity metrics, community composition, and assembly processes, we aimed to address the following scientific questions: (1) How do soil physicochemical properties evolve over a millennium of reclamation? (2) How do the abundance, diversity, and community structure of archaea and bacteria respond to long-term reclamation? (3) What are the respective roles of deterministic and stochastic processes in the assembly of archaeal and bacterial communities, and how do these processes shift with reclamation years? and (4) What are the relative contributions of environmental factors, geographic distance, and reclamation years to microbial biogeographic patterns?

Based on the contrasting ecological strategies and environmental adaptability of archaea and bacteria reported in previous studies [17,21], and the profound changes in soil properties induced by reclamation, we formulated the following hypotheses: (1) Archaeal and bacterial abundances and α-diversity will exhibit distinct temporal patterns along the reclamation chronosequence, with archaeal abundance remaining relatively stable due to their adaptation to saline-alkaline reclamation conditions, while bacterial diversity will show a more pronounced increase during mid-term reclamation stages (e.g., 210 years) due to improved nutrient availability and soil structure; (2) Archaeal community assembly will be predominantly governed by stochastic processes reflecting their broader environmental tolerance, whereas bacterial community assembly will be increasingly dominated by deterministic processes as reclamation proceeds; and (3) Soil pH and electrical conductivity (EC) will be the primary environmental drivers shaping microbial community composition, with bacterial communities showing stronger distance–decay relationships along environmental gradients than archaeal communities.

By elucidating the long-term ecological succession of soil microbial communities driven by coastal reclamation, this study provides new insights into soil ecosystem development and offers valuable scientific guidance for farmland management and ecological restoration in reclaimed coastal regions.

## 2. Materials and Methods

### 2.1. Soil Samples Collection

The study area is located on the southern shore of Hangzhou Bay in Cixi, Zhejiang Province, China, within the northern subtropical monsoon climate zone (30°02′–30°24′ N, 121°02′–121°42′ E). In 2021, the region had an annual mean temperature of 18.5 °C and 1515.4 h of sunshine, showing typical monsoon climatic characteristics. The mean annual precipitation is 2161.1 mm. The area includes a 77 km arcuate coastline with the fastest sedimentation rate in Hangzhou Bay. Since AD 1047, large-scale coastal reclamation has been conducted, each with well-documented construction years.

**Figure 1:**
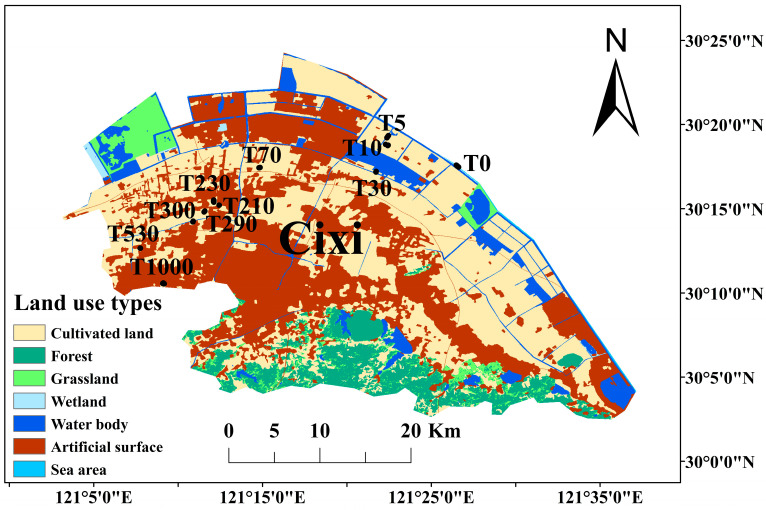
Location of collected cultivated choronosequence soils and coastal salt marsh.

Soil samples were collected in December 2021 from dryland agricultural fields located in 10 reclaimed zones of different reclamation ages along a transect perpendicular to the coastline. Over the past 20 years, these fields have been managed under a mung bean–broccoli rotation, with broccoli grown in the sampling season. Coastal tidal flat soil was used as the unreclaimed control, with a reclamation age of 0 years (T0). The other reclaimed sites were assigned reclamation years based on their construction years: 5 (T5), 10 (T10), 30 (T30), 70 (T70), 210 (T210), 230 (T230), 290 (T290), 300 (T300), 530 (T530), and 1000 (T1000) years. For sampling sites with reclamation years of 30 to 1000 years, the age is calculated based on the initial construction time of the local seawalls as recorded in the county’s historical records. For sampling sites with reclamation years of 5 and 10 years, the initial construction time of the new seawalls is obtained by consulting relevant experts from the Ningbo Academy of Agricultural Sciences. At each site, 6 replicate samples were collected using a five-point sampling method at a depth of 0–20 cm, with distances between sampling points exceeding 100 m (Figure 1). Visible stones and roots were removed immediately. A portion of each soil sample was air-dried for physicochemical analyses, while the remaining portion was stored at −80 °C for subsequent microbial analyses.

### 2.2. Determination of Soil Physicochemical Properties

Soil physicochemical properties were measured following the procedures described in Soil Agricultural Chemical Analysis [22]. Soil pH was determined using a glass electrode. Electrical conductivity (EC) was measured using the conductivity method. Total organic carbon (TOC) was quantified using a total organic carbon analyzer (Multi N/C 3100, Analytik Jena, Jena, Germany). Recalcitrant carbon (RC) was determined using acid hydrolysis. Total nitrogen (TN) was analyzed using the semi-micro Kjeldahl digestion method. Alkali-hydrolysable nitrogen (AN) was determined using the diffusion method. Available phosphorus (AP) was measured using the Olsen-P method followed by molybdenum-antimony colorimetry. Available potassium (AK) was extracted with ammonium acetate. Cation exchange capacity (CEC) was determined using the ammonium acetate exchange method. Soil particle-size composition (silt-clay fraction, PP; sand fraction, SP) was analyzed with a laser particle size analyzer.

### 2.3. Soil DNA Extraction, PCR Amplification and Illumina High Throughput Sequencing

Total DNA was extracted from soil using the FastDNA Spin Kit for Soil (MP Biomedicals, Irvine, CA, USA). The archaea and bacteria 16S rRNA genes were amplified using prokaryotic 16S rRNA gene universal primers 515F (5′-GTGCCAGCMGCCGCGGTAA-3′) and 909R (5′-CCCCGYCAATTCMTTTRAGT-3′) [23]. PCR reactions (25 μL total volume) were performed in triplicate, containing 12.5 μL 2× EasyTaq PCR SuperMix (TransGen Biotech, Beijing, China), 0.4 μL of each primer (10 μM), 1 μL tenfold diluted DNA template, and 10.7 μL molecular biology-grade water. PCR products were verified by 1.5% agarose gel electrophoresis, purified with AxyPrep^®^ DNA Gel Extraction Kit (Axygen Biosciences, Union City, CA, USA). Purified amplicons were quantified with a Qubit 4.0 Fluorometer (Thermo Fisher Scientific, Waltham, MA, USA) and pooled in equimolar concentrations. Sequencing libraries were constructed and paired-end sequenced on an Illumina HiSeq 2500 platform (Illumina, San Diego, CA, USA). Sequencing data were processed using QIIME v1.8.0. Raw reads were demultiplexed, and low-quality sequences were filtered by removing those with: (1) length < 150 bp, (2) average Phred score < 20, (3) ambiguous bases, or (4) mononucleotide repeats > 8 bp [24]. Paired-end reads were merged with FLASH v1.2.7, followed by chimera removal. High-quality sequences were clustered into OTUs at 97% identity using UCLUST feature in QIIME v1.8.0. Taxonomic assignment was performed by BLAST feature in QIIME v1.8.0 alignment against the Silva database (138.2) with 90% confidence cutoff. Data were sparsified to 90% of the minimum sequencing depth by 100 iterations of resampling to generate a standardized OTU table for subsequent analysis. All sequence reads have been submitted to the Sequence Read Archive (SRA) database of the National Center for Biotechnology Information (NCBI) under the accession numbers PRJNA1371700.

### 2.4. Fluorescence-Based Quantitative PCR

For any soil DNA sample, the 16S rRNA gene fragments of Archaea and Bacteria were amplified using the primers Arch 967F (5′-AATTGGCGGGGGAGCAC-3′) and Arch-1060R (5′-GGCCATGCACCWCCTCTC-3′) [25], as well as Bacterial Eub338 (5′-ACTCCTACGGGAGGCAGCAG-3′) and Eub518 (5′-ATTACCGCGGCTGCTGG-3′) [26]. The amplification system included: 25 μL of 2× Taq Master Mix, 1 μL of each upstream and downstream primer, 2 μL of total soil DNA, and 21 μL of ddH_2_O. After amplification, the products were analyzed by 0.75% agarose gel electrophoresis, and the bands were excised for purification using the BioTeke DNA Gel Recovery Kit.

### 2.5. Statistical and Bioinformatic Analysis

All statistical analyses were conducted using R (version 4.3.3). Differences among variables were assessed using SPSS 25. Alpha diversity was calculated using the “vegan” package in R. Unweighted Pair-Group Method with Arithmetic Mean (UPGMA), a widely used hierarchical clustering approach, was performed with the “ggtree” package to cluster microbial communities at both the phylum and genus levels. Mantel tests were employed to evaluate correlations between two distance matrices, and significance was assessed through permutation tests [27].

Phylogenetic null model analysis was carried out to quantify the relative contributions of deterministic and stochastic processes to microbial community assembly. Beta mean nearest taxon distance (βMNTD) was calculated using the function “comdistnt” in the “picante” package, and the standardized β-nearest taxon index (βNTI) was subsequently derived. Based on established thresholds, βNTI < −2 indicates homogeneous selection as the dominant deterministic process; βNTI > 2 suggests variable selection; and −2 < βNTI < 2 reflects a predominance of stochastic processes in community assembly [28].

Distance decay relationships (DDR) were evaluated using Bray–Curtis dissimilarity to quantify community similarity among samples. Geographic distance was computed using the Haversine formula, and Euclidean distances of soil physicochemical variables were calculated with the “vegan” package. Differences in reclamation years were derived from pairwise absolute differences, and their correlations with microbial community dissimilarity were assessed using Mantel tests with Spearman coefficients.

The variance inflation factor (VIF) analysis was performed using the “car” package to examine multicollinearity among environmental variables. Variance Partitioning Analysis (VPA) was conducted to determine the proportion of community variation explained by environmental factors, geographic distance, and reclamation years, using the “vegan”package. Environmental variables were further categorized into four groups based on their functional characteristics: pH and electrical conductivity (pH + EC), soil carbon components (TOC + RC), soil nitrogen, phosphorus, and potassium contents (TN + AN + AP + AK), and soil particle-size fractions (PP + SP). VPA was then performed for each environmental group independently.

## 3. Results

### 3.1. Soil Physicochemical Properties of the Topsoil Layer

**Figure 2:**
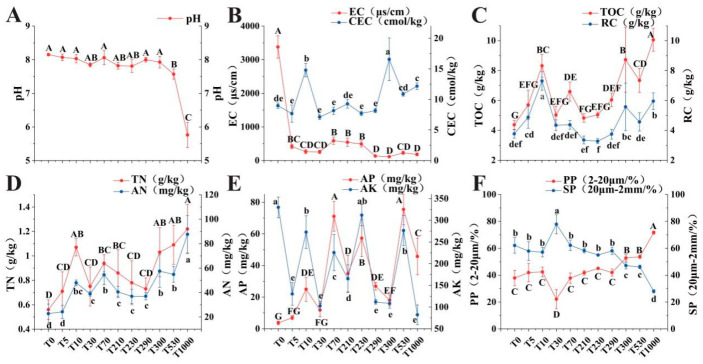
Physicochemical properties of the plow layer soil under different reclamation years. (**A**) pH; (**B**) EC (Electrical Conductivity) and CEC (Cation Exchange Capacity); (**C**) TOC (Total Organic Carbon) and RC (Retained Carbon); (**D**) TN (Total Nitrogen) and AN (Alkali-hydrolyzable Nitrogen); (**E**) AP (Available Phosphorus) and AK (Available Potassium); (**F**) PP (Powder Particle) and SP (Sand Particle). Letters indicate significant differences among reclamation years for each property (*p* < 0.05). Properties marked in red (pH, EC, TOC, TN, AP, PP) are annotated with uppercase letters; properties marked in blue (CEC, RC, AN, AK, SP) are annotated with lowercase letters.

There were significant differences in the physicochemical properties of the topsoil cultivation layer under different reclamation years (Figure 2, Table S1). Except for the 1000-year reclaimed soil, which was acidic, soils reclaimed for 0–530 years were all alkaline. The EC of the coastal soil was much higher than that of the reclaimed soils, indicating a very high salt content. The TOC and TN contents were lowest in the coastal soil and increased after reclamation due to human activities such as fertilization. During the initial 0–30 years of reclamation, the proportion of RC in TOC was relatively high, but this proportion began to decrease as the reclamation year extended. The AN content was lowest in the coastal soil and increased after reclamation. The AP content was very low in both coastal soil and 5-year reclaimed soil. Potassium ion is one of the important ions constituting soil salinity. The coastal soil had high salinity, hence its AK content was also high. As the soil was reclaimed for farmland, the decrease in salinity led to the leaching of potassium ions, resulting in a subsequent decrease in AK content. Regarding soil texture, except for some samples containing a small amount of clay (<2 μm), most samples were composed of silt (2–20 μm) and sand (20 μm–2 mm). The soil reclaimed for 30 years had the highest sand content, while the soil reclaimed for 1000 years had the highest silt content. With increasing reclamation year, soil particles showed a trend towards becoming finer.

From a microbiological perspective, soil environmental heterogeneity is considered to promote species coexistence and richness. As shown in Figure S1, overall, there was no significant linear relationship between environmental heterogeneity and reclamation year. Environmental heterogeneity first increased and then decreased during the 5–70 years of reclamation, peaking at 10 years and reaching its lowest point at 70 years; for the remaining reclamation years, environmental heterogeneity remained relatively stable.

### 3.2. Changes in Prokaryotic Microbial Abundance and Diversity

**Figure 3:**
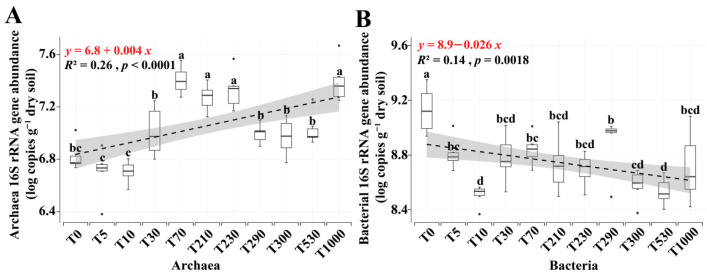
Abundance of archaeal and bacterial communities in the plow layer soil under different reclamation years. ((**A**) Archaeal abundance; (**B**) Bacterial abundance). Various lowercase letters indicate significant differences among reclamation years (*p* < 0.05).

Figure 3A,B shows the 16S rRNA gene copy numbers of archaea and bacteria in the topsoil cultivation layer under different reclamation years. After log10 transformation, the gene copy numbers of archaea ranged from 6.70 to 7.40, while those of bacteria ranged from 8.51 to 9.13. Overall, bacterial gene copy numbers were two orders of magnitude higher than those of archaea. The archaeal gene copy numbers were relatively low during the early 0–10 years of reclamation and higher during the 70–230 year period. Bacterial gene copy numbers were highest in the coastal soil, continuously decreased during the 5–10 year reclamation period, and remained relatively stable from 30 to 1000 years of reclamation.

Table S2 analyzes the correlations between physicochemical factors and the gene copy numbers of archaea and bacteria in the topsoil cultivation layer. Fewer physicochemical factors affected archaeal gene copy numbers, with only pH, AN, and AP showing significant effects. In contrast, many physicochemical factors significantly influenced bacterial gene copy numbers; except for AK, all other measured factors had a significant impact. pH showed a significant negative correlation with archaeal gene copy numbers, while AN and AP showed significant positive correlations. For bacterial gene copy numbers, pH, EC, and SP showed significant positive correlations, whereas TOC, RC, TN, AN, AP, and PP showed significant negative correlations.

For both archaea and bacteria, the number of OTUs in the coastal soil was much lower than in the reclaimed soils. For the archaeal community (Table 1), the OTU, Chao1, and ACE indices all showed a trend of first increasing and then decreasing with increasing reclamation year, reaching their highest values at 210 and 230 years of reclamation. The Shannon and Simpson indices, however, fluctuated over time, reaching their maximum at 290 years and their minimum at 1000 years of reclamation. For the bacterial community (Table 2), the OTU, Chao1, and ACE indices also showed a trend of first increasing and then decreasing with reclamation years, peaking at 210 years. The Shannon and Simpson indices were much lower in the coastal soil compared to the reclaimed soils.

### 3.3. Structural Shifts in Prokaryotic Microbial Communities and Their Environmental Determinants

**Figure 4:**
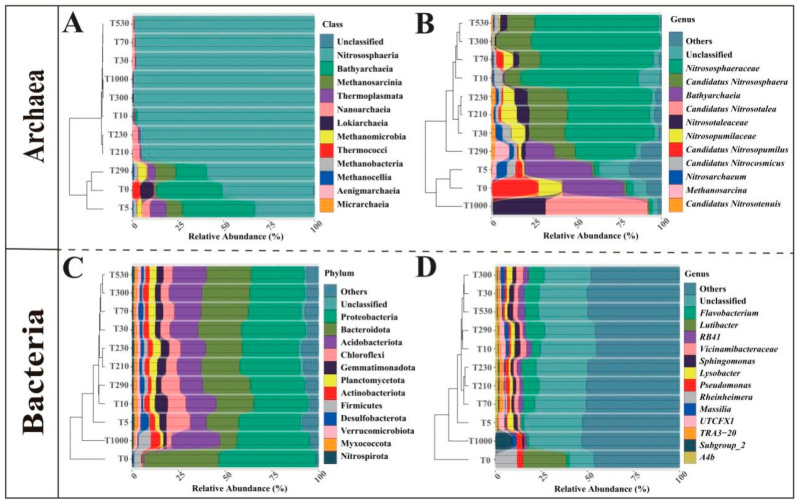
Community composition of the archaeal and bacterial in the plow layer soil under different reclamation years, with a clustering tree for each treatment. ((**A**) Archaeal class-level composition; (**B**) Archaeal genus-level composition; (**C**) Dominant bacterial phylum-level composition; (**D**) Dominant bacterial genus-level composition).

A total of 12 archaeal classes and 52 bacterial phyla were identified from all plow layer soil samples. Figure 4A,C display all archaeal classes and the dominant bacterial phyla (relative abundance > 1%), respectively. Significant differences in community composition were observed between coastal and reclaimed soils. For archaea (Figure 4A), Nitrososphaeria was the class with the highest mean relative abundance, exceeding 95% in all soils except those at 0, 5, and 290 years of reclamation. This was followed by Bathyarchaeia, which was more abundant in soils at 0, 5, and 290 years of reclamation. Methanosarcina and Thermoplasmata were predominantly found in soils at 5 and 290 years, while Lokiarchaeia and Thermococci were primarily distributed in coastal soils. For bacteria (Figure 4C), Proteobacteria and Bacteroidota consistently showed high relative abundances across all samples and dominated in coastal soils. Following the reclamation of coastal soils for agriculture, the proportions of Proteobacteria and Bacteroidota decreased, while the proportions of other phyla, such as Acidobacteriota and Chloroflexi, increased.

Overall, 39 archaeal genera and 994 bacterial genera were identified from all OTUs. The dominant genera (relative abundance > 1%) are shown in Figure 4B,D. Among archaeal genera (Figure 4B), *Nitrososphaeraceae* and *Candidatus Nitrososphaera* were highly abundant in all soils except those at 0, 5, and 1000 years of reclamation. *Bathyarchaeia* was mainly distributed in soils at 0, 5, and 290 years of reclamation. *Candidatus Nitrosotalea* was abundant exclusively in the 1000-year reclaimed soil. The abundance of *Nitrosotaleaceae* was low in unreclaimed and short-term reclaimed soils, began to increase after 30 years of reclamation, and reached its maximum at 1000 years. Among bacterial genera (Figure 4D), *Lutibacter* and *Rheinheimera* constituted a very large proportion in coastal soils but were almost absent in reclaimed soils; these genera are primarily found in freshwater and seawater. In reclaimed soils, *Flavobacterium* was the most dominant genus, its proportion showing an initial increase followed by a decreasing trend with the reclamation years.

With the extension of reclamation years, the composition of archaeal and bacterial communities undergoes changes, thereby further leading to alterations in community functions (Figure S2). The functions of Aerobic_ammonia_oxidation and Nitrification in archaeal communities are significantly enhanced in reclaimed soils. The functions of Chemoheterotrophy and Aerobic_chemoheterotrophy account for a relatively high proportion in bacterial communities across all reclamation years, while the functions of thiosulfate_respiration, dark_sulfite_oxidation, and dark_sulfur_oxidation show a sharp decline after reclamation.

NMDS analysis based on the OTU level revealed that both archaeal and bacterial communities changed along the reclamation chronosequence (Figure S3). The archaeal communities from soils at 0, 5, 10, 290, and 1000 years of reclamation showed clear separation (Figure S3A), whereas among bacterial communities, only samples from 0, 5, and 1000 years were distinctly separated (Figure S3B). Substantial shifts in both archaeal and bacterial communities occurred during the initial stage (0–5 years) and at 1000 years of reclamation, while the communities changed less during the intermediate stages. The number of years with minimal community change was fewer for archaea than for bacteria.

### 3.4. Assembly Processes of Prokaryotic Microbial Communities

**Figure 5:**
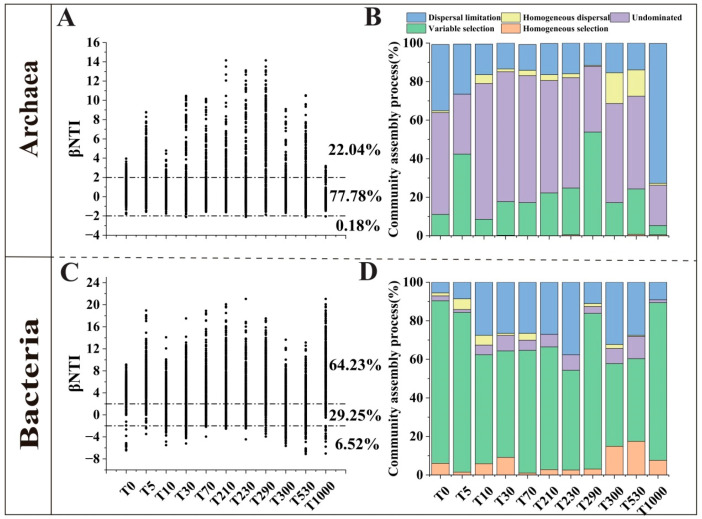
βNTI values and assembly processes of archaeal and bacterial communities in the plow layer soil under different reclamation years. ((**A**) Archaeal βNTI values; (**B**) Archaeal assembly processes; (**C**) Bacterial βNTI values; (**D**) Bacterial assembly processes).

To investigate the spatiotemporal succession in the phylogenetic composition of prokaryotic microbial communities, the Beta Nearest Taxon Index (βNTI) was calculated for both archaeal and bacterial communities. This was combined with the RCbray value to determine the proportion of community assembly processes (Figure 5). The archaeal communities were overall dominated by stochastic processes (77.78%), with only 22.22% of the community turnover attributed to deterministic processes (Figure 5A). Among the stochastic processes, undominated processes were the most prevalent, followed by dispersal limitation and a small contribution from homogenizing dispersal. The influence of dispersal limitation generally decreased over the 0–530 years of reclamation but increased sharply at the 1000-year mark (Figure 5B). The βNTI values of archaeal communities showed significant linear relationships with geographic distance, environmental distance, and the difference in reclamation years, decreasing as these three factors increased (Figure S4A–C).

In contrast, bacterial communities were primarily governed by deterministic processes (70.75%), with only 29.25% of the turnover due to stochastic processes (Figure 5C). Variable selection was the dominant deterministic process. During the first 0–230 years of reclamation, the relative influence of variable selection decreased with increasing reclamation years, but it increased abruptly after 290 years, then decreased again, and finally increased once more at 1000 years. Homogeneous selection showed an increasing trend with reclamation years during two periods: 5–30 years and 70–530 years (Figure 5D). The βNTI values of bacterial communities also exhibited significant linear relationships with geographic distance, environmental distance, and the difference in reclamation years. However, unlike the archaeal communities, the bacterial βNTI values increased as these three factors increased (Figure S4D–F).

### 3.5. Contributions of Geographical Distance and Environmental Factors to Prokaryotic Microbial Community Variation

**Figure 6:**
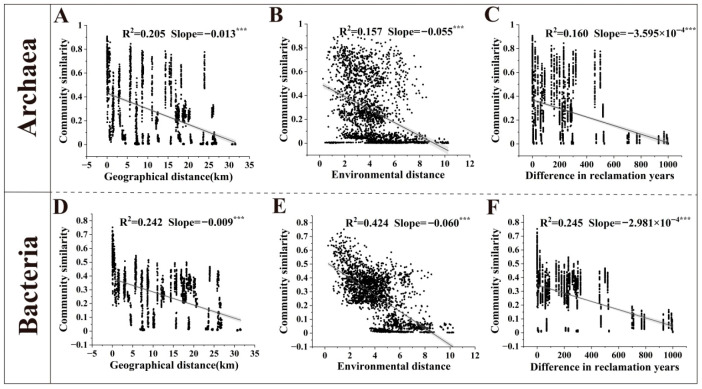
Similarity of archaeal and bacterial communities in the plow layer soil in relation to geographic distance, environmental distance, and differences in reclamation years. ((**A**) Archaeal community similarity vs. geographic distance; (**B**) Archaeal community similarity vs. environmental distance; (**C**) Archaeal community similarity vs. difference in reclamation years; (**D**) Bacterial community similarity vs. geographic distance; (**E**) Bacterial community similarity vs. environmental distance; (**F**) Bacterial community similarity vs. difference in reclamation years; *** *p* < 0.001).

Based on distance-decay relationship models, we analyzed the effects of geographical distance, environmental distance, and the difference in reclamation years on the similarity of archaeal and bacterial communities (Figure 6). The results demonstrated that all three factors exhibited highly significant negative correlations with both archaeal and bacterial community similarity (*p* < 0.001), indicating a typical distance-decay pattern. The correlations between these factors and bacterial communities were stronger than those with archaeal communities, suggesting that bacterial communities varied more substantially than archaeal communities. For archaeal communities, geographical distance showed the strongest correlation, whereas for bacterial communities, environmental distance was the most strongly correlated.

Multiple Regression on Similarity Matrices (MRM) was used to quantify the relative influence of different factors on microbial communities (Table S3). The models for archaeal and bacterial communities explained 50% and 75% of the variation in community similarity, respectively (*p* = 0.0001), indicating that bacteria are more influenced by environmental factors than archaea. Specifically, geographical distance, pH, RC, and AN showed significant negative correlations with both archaea and bacteria. Among these, pH had the strongest correlation, and its correlation with archaea was stronger than with bacteria. TOC showed significant positive correlations with both archaea and bacteria, also exerting a stronger influence on archaea. Soil particle size strongly affected bacterial community similarity. As the reclaimed soils consisted almost entirely of silt and sand, these two factors showed completely opposite effects: as silt content decreased and sand content increased, bacterial community similarity gradually decreased.

**Figure 7:**
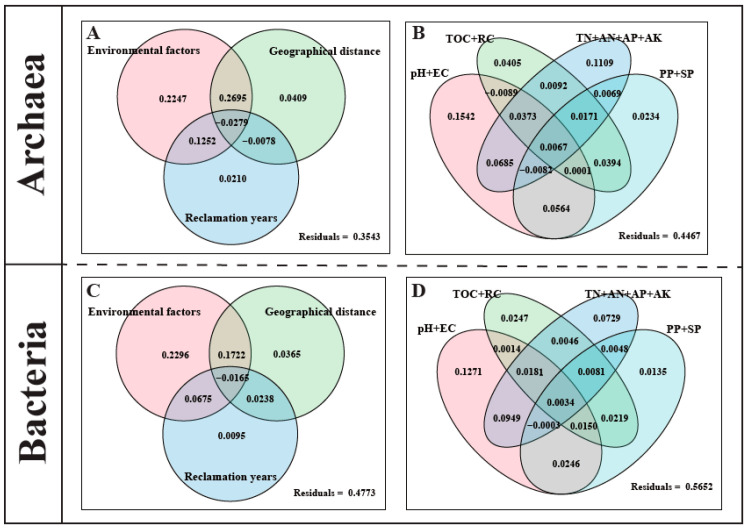
Variance partitioning analysis (VPA) of factors influencing the variation in archaeal and bacterial community structures in the plow layer soil. ((**A**): VPA of the contribution of environmental factors, geographic distance, and reclamation years to changes in community structure for archaea; (**B**): VPA of the contribution of different soil properties combinations to changes in community structure for archaea; (**C**): VPA of the contribution of environmental factors, geographic distance, and reclamation years to changes in community structure for bacteria; (**D**): VPA of the contribution of different soil properties combinations to changes in community structure for bacteria).

Variance Partitioning Analysis (VPA) was performed to determine the explanatory power of different factors for microbial community variation (Figure 7A,C). Environmental factors, geographical distance, and reclamation years together explained 64.57% of the variance in archaeal communities and 52.27% in bacterial communities. Among these, environmental factors alone had the highest explanatory power (22.47% for archaea, 22.96% for bacteria), followed by geographical distance alone (4.09% for archaea, 3.65% for bacteria). Reclamation years alone explained only 2.10% and 0.95% of the variance for archaea and bacteria, respectively. When environmental factors were divided into four groups, they collectively explained 55.33% of the variance in archaeal communities and 43.48% in bacterial communities (Figure 7B,D). Among these groups, pH plus EC had the highest individual explanatory power, followed by nitrogen and phosphorus content (TN + AN + AP + AK). Organic carbon content (TOC + RC) and soil particle size (PP + SP) had relatively low individual explanatory power. The variance inflation factor (VIF) analysis results showed that the VIF values for pH and EC were 4.70 and 3.70, respectively, both below the threshold of 5, indicating a low level of multicollinearity. As key environmental factors influencing community variation, pH and EC demonstrate independent effects.

## 4. Discussion

### 4.1. Evolution of Soil Physicochemical Properties and Microbial Abundance Driven by Reclamation

Our findings demonstrate that long-term reclamation has profoundly altered the soil’s physicochemical environment. The soils exhibited a clear trend of desalination and eutrophication, characterized by increasing levels of TOC, TN, and AN, alongside decreasing pH and EC (Figure 2), consistent with previous studies [9,10]. The decline in pH is primarily driven by pyrite oxidation [29] and the decomposition of organic matter [30], while the sharp decrease in EC during the initial reclamation stages is mainly attributed to the leaching of carbonates (decalcification) [31]. However, soil nutrient status did not improve continuously. AP and AK decreased over the long term (e.g., 70–300 and 530–1000 years) after an initial short-term increase, providing evidence for phosphorus and other available nutrient limitations during soil development [32]. Concurrently, soil texture changed, with particles gradually becoming finer and sand transforming into silt as reclamation years increased (Figure 2F). A study reported a decrease in the mean particle size of surface soil (20 cm) after 35 years of reclamation compared to 3 years in Fengxian, Yangtze Estuary [33]. Furthermore, changes in soil texture are not merely adjustments in physical structure but also a critical factor regulating microbial habitats and functions. This study observed that with increasing reclamation years, soil particles gradually become finer. This alteration in texture directly influences soil pore structure, aeration, and water-holding capacity, thereby affecting the composition and abundance of microbial communities. Similarly, Ma et al. found in their study on different land use types that native grassland (NG) exhibited higher silt content and lower sand content, which were significantly correlated with greater soil porosity and organic carbon accumulation [34]. This provides a more suitable habitat for microorganisms.

The evolution of these physicochemical properties directly drives the different responses of microbial abundance in the plow layer soil to the reclamation years. During the initial stage of reclamation (0–10 years), drastic environmental changes (such as desalination) led to the death of a large number of marine-origin bacteria (e.g., *Lutibacter*, *Rheinheimera*), resulting in a significant reduction in bacterial abundance. In contrast, archaeal communities, particularly ammonia-oxidizing archaea, exhibit greater tolerance to fluctuating saline-alkaline conditions and may possess metabolic plasticity that buffers them against early environmental stress [21]. Starting from around 30 years of reclamation, with continuous nutrient input and improved soil conditions, the abundance of both archaea and bacteria begins to increase in a synchronized manner [9,35].

Correlation analyses between microbial abundance and soil physicochemical factors revealed their distinct ecological preferences. Archaeal abundance correlated negatively with pH but positively with AN and AP, indicating a preference for neutral soils rich in available nutrients. In contrast, bacterial abundance showed positive correlations with pH and EC but negative correlations with various nutrient elements (Table S2). While most bacteria generally prefer nutrient-rich and low-salinity soils [36], the seemingly paradoxical pattern observed here may stem from the high abundance of marine-adapted bacteria tolerant of high pH and salinity in the pre-reclamation coastal soil. The subsequent reduction in soil salinity and alkalinity following reclamation likely led to the decline of these specialized bacterial populations, thereby shaping this unique response pattern.

### 4.2. Reclamation Induces Significant Shifts in Soil Archaeal and Bacterial Community Diversity and Composition

Reclamation of coastal wetlands significantly altered the composition and diversity of soil archaeal and bacterial communities. In the plow layer, the observed diversity indices of both archaea and bacteria generally increased after reclamation (Table 1 and Table 2). This can likely be attributed to the decrease in soil pH and salinity, along with improved nutrient conditions, which collectively provided a more suitable habitat for a wider range of microbial taxa [37,38].

Within the archaeal community, the class Nitrososphaeria dominated (Figure 4A). Most members of this group are ammonia-oxidizing archaea that prefer freshwater environments [39]. Their relative abundance increased significantly in reclaimed soils. Similarly, Arunrat et al. also found in their study on shifting cultivation systems in northern Thailand that Nitrososphaerota (the phylum to which Nitrososphaeria belongs) was significantly enriched in soils recently subjected to slash-and-burn and tillage treatments, and showed a significant positive correlation with soil electrical conductivity (ECe) [40]. The study further pointed out that the enrichment of ammonia-oxidizing archaea, such as *Nitrosocosmicus* and *Nitrososphaera,* reflects an enhanced potential for nitrogen transformation in the system after disturbance, particularly in the ammonia oxidation process. This aligns with the pattern observed in the present study, where these taxa showed higher abundance in reclaimed soils. From a functional perspective, the enrichment of Nitrososphaeria signifies a shift in the archaeal community towards nitrogen-cycling functional types. In reclaimed soils, ammonia-oxidizing archaea drive the upstream reaction of nitrification by converting ammonium to nitrite, thereby influencing the rate and fate of the entire nitrogen cycle [40].

The class Bathyarchaeia, commonly found in marine sediments [41], was relatively abundant in unreclaimed and early-stage reclaimed soils. In contrast, Lokiarchaeia and Thermococci, primarily associated with deep-sea hydrothermal vent sediments [42,43], were only detected in some coastal soils. At the genus level, *Nitrososphaeraceae* and *Candidatus Nitrososphaera* (both belonging to the family Nitrososphaeraceae) were dominant in all soils except those at 0, 5, and 1000 years of reclamation. In contrast, *Candidatus Nitrosotalea* and *Nitrosotaleaceae* (family Nitrosotaleaceae) reached their highest relative abundance in soils reclaimed for 1000 years, which were acidic (Figure 4B). This is consistent with findings by Zhao et al. [44], indicating that ammonia-oxidizing archaea within the order Nitrososphaerales prefer alkaline soil conditions, while those in the family Nitrosotaleaceae are more adapted to acidic environments.

In the bacterial community, the phylum Proteobacteria was the most dominant across all reclamation stages (Figure 4C), consistent with previous studies [45]. Unreclaimed coastal soils were largely dominated by the phylum Bacteroidota, mainly due to the high relative abundance of halotolerant genera such as *Lutibacter* [46], resembling microbial community structures found in marine sediments [47]. After reclamation, the relative abundances of Acidobacteriota, Chloroflexi, and Gemmatimonadota increased significantly. The first two are commonly found in rice rhizospheres [48,49] and are sensitive to soil pH [50]. Gemmatimonadota, on the other hand, prefers neutral and relatively dry soil conditions [51]. This suggests that root exudates and soil acidification may have contributed to their increase. At the genus level, typical coastal taxa such as *Lutibacter* and *Rheinheimera* [52,53] nearly disappeared after reclamation. In contrast, genera involved in soil nutrient cycling—such as *Flavobacterium*, *RB41*, *Vicinamibacteraceae*, *Sphingomonas*, and *Lysobacter*—became dominant in reclaimed soils (Figure 4D), suggesting their important roles in regulating nutrient dynamics in these environments [54].

### 4.3. Divergent Assembly Mechanisms of Archaeal and Bacterial Communities After Land Reclamation

Analysis of community assembly processes is a crucial approach for elucidating the dynamics of microbial communities [19,55]. Deterministic processes emphasize the shaping of community structure by biotic or abiotic factors, whereas stochastic processes reflect unpredictable dynamics caused by probabilistic dispersal and random disturbances [56,57]. This study revealed that the assembly of archaeal communities was predominantly governed by stochastic processes, with a notable dominance of entirely random patterns. In contrast, bacterial communities were primarily influenced by deterministic processes, mainly through homogeneous selection (Figure 5). Previous research has indicated that archaea exhibit stronger environmental adaptability to saline-alkali soils [21], making their communities less susceptible to environmental changes and more inclined toward stochastic succession. Bacteria, however, are more sensitive to nutrient conditions and prone to environmental filtering.

Compared to pristine coastal soils, the role of homogeneous selection in archaeal community assembly increased after reclamation, while dispersal limitation decreased. However, this trend reversed at the 1000-year reclamation stage, showing extremely high dispersal limitation and very low homogeneous selection (Figure 5B). This shift may be attributed to tillage practices disrupting soil aggregate structures, thereby facilitating the spatial dispersal of archaea [58]. The soil reclaimed for 1000 years exhibited a significantly different pH compared to soils of other reclamation years (with a pH of 5.76, indicating acidity, while soils of other reclamation years were alkaline). This led to a substantial change in its community composition (Figure 4B), thereby limiting the diffusion capacity of archaea. For bacterial communities, the assembly process showed little change at the 5-year reclamation stage. However, during the 10–230 and 300–530-year stages, homogeneous selection weakened while dispersal limitation intensified, possibly due to similar soil environmental conditions and anthropogenic disturbance patterns across these periods. The exceptionally high homogeneous selection observed in the 290 and 1000-year reclaimed soils may result from significant variations in specific environmental factors (Figure 5D). Moreover, increasing geographical distance enhances phylogenetic turnover, which can also strengthen deterministic processes in bacterial communities [59]. In this study, the βNTI values of archaeal communities showed significant negative correlations with geographical distance, environmental distance, and the difference in reclamation years (*p* < 0.001), whereas bacterial βNTI values exhibited significant positive correlations with these factors (*p* < 0.001), further confirming the fundamental differences in their assembly mechanisms (Figure S4).

### 4.4. Environmental Filtering and Spatial Dispersal Shape the Geographical Patterns of Prokaryotic Microorganisms

Bacterial communities exhibit steeper distance-decay relationships than archaeal communities, with a particularly heightened sensitivity to environmental gradients (Figure 6), a pattern likely driven by multiple factors. This divergence fundamentally stems from their distinct ecological strategies: bacterial communities typically respond more strongly to environmental filtering (e.g., pH, salinity, nutrients), resulting in composition that varies markedly with landscape heterogeneity, whereas archaea, due to their physiological adaptations to saline-alkaline and other extreme conditions, maintain broader and more stable distributions [60]. Furthermore, landscape-level dispersal limitations amplify this difference: bacteria may be more constrained by geographical barriers (e.g., mountains, vegetation) or disruptions in hydrological connectivity, leading to geographical isolation, while archaea may possess a greater potential for long-distance dispersal, thereby weakening spatial decay [61]. The legacy effects of historical land use also play a significant role, as past disturbances such as agricultural activities may exert more persistent impacts on bacterial communities [62], consequently intensifying the slope of the distance-decay relationship.

MRM showed that environmental factors explained a significantly higher proportion of the variation in bacterial communities (75%) than in archaeal communities (50%). pH, organic carbon, recalcitrant carbon, and available nitrogen were key factors jointly influencing the community structures of both archaea and bacteria, while soil particle size specifically had a significant impact only on bacterial communities (Table S3). This aligns with previous studies indicating that pH, organic matter, and soil texture collectively shape prokaryotic microbial communities [63,64,65]. However, the explanatory patterns derived from VPA differed from those of MRM: the explained variation was slightly higher for archaeal communities than for bacterial communities (64.57% vs. 52.27%) (Figure 7). This discrepancy may stem from the substantial “unexplained variation” inherent in the VPA model, which is often attributed to stochastic ecological processes such as growth, death, colonization, and extinction [66]. These unexplained variations may also originate from some unmeasured environmental factors, such as redox potential, metal elements, organic matter composition, and others. For both domains, the individual effect of environmental factors and their joint effect with geographical distance accounted for the highest proportions of explained variation, indicating that the effect of environmental filtering outweighed the effects of pure spatial dispersal limitation (geographical distance) or temporal succession (the difference in reclamation years). When environmental factors were grouped for analysis, the combination of pH and electrical conductivity (often used as an indicator of salinity) had the highest individual explanatory power for both archaeal and bacterial communities (Figure 7B,D), robustly confirming their role as the primary environmental drivers of microbial geographic distribution [63,67].

## 5. Conclusions

Coastal reclamation profoundly reshapes soil physicochemical properties and drives long-term ecological succession of prokaryotic communities in the plow layer. Along the 0–1000-year reclamation chronosequence, soils exhibited continuous desalination, acidification, nutrient accumulation, and particle-size refinement. These changes in soil properties led to distinct temporal patterns in archaeal and bacterial abundance and diversity, with both groups reaching peak α-diversity at approximately 210–230 years. Reclamation also triggered significant shifts in community composition, including the replacement of marine-derived taxa by terrestrial nutrient-cycling microorganisms. Archaea and bacteria displayed fundamentally different ecological assembly mechanisms. Archaeal communities were predominantly governed by stochastic processes—mainly undominated processes and dispersal limitation—reflecting their broad environmental adaptability. In contrast, bacterial communities were primarily structured by deterministic processes, particularly variable selection, indicating stronger sensitivity to environmental gradients. Distance–decay patterns further revealed that bacteria were more responsive to environmental factors, whereas archaeal communities exhibited higher spatial turnover. Environmental factors, especially pH and EC, were the key drivers shaping community composition. Overall, this study comprehensively elucidates how long-term coastal land reclamation rebuilds soil archaeal and bacterial communities, and provides important insights for sustainable farmland management and ecological restoration in reclaimed agricultural soils.

## Figures and Tables

**Table 1:** Community diversity of archaea in plow layer soils with different reclamation years.

Sample	OTU	Chao1	ACE	Shannon	Simpson
T0	18 ± 3 g	47.24 ± 20.28 de	36.33 ± 7.67 d	2.57 ± 0.12 b	0.897 ± 0.015 ab
T5	56 ± 6 cd	69.11 ± 11.97 bcd	66.95 ± 8.66 c	3.34 ± 0.16 a	0.939 ± 0.015 a
T10	48 ± 11 de	59.67 ± 15.23 cd	65.20 ± 15.13 c	2.01 ± 0.29 c	0.756 ± 0.070 cd
T30	63 ± 7 bc	89.15 ± 9.83 b	87.70 ± 10.55 b	2.44 ± 0.17 b	0.797 ± 0.058 bc
T70	70 ± 7 b	86.27 ± 15.69 b	86.22 ± 14.94 b	1.96 ± 0.41 cd	0.638 ± 0.130 e
T210	93 ± 6 a	110.16 ± 10.09 a	115.94 ± 9.81 a	2.68 ± 0.23 b	0.827 ± 0.054 bc
T230	94 ± 11 a	109.98 ± 16.59 a	111.93 ± 11.87 a	2.70 ± 0.23 b	0.837 ± 0.046 abc
T290	67 ± 7 bc	76.54 ± 9.98 bc	77.13 ± 10.95 bc	3.44 ± 0.16 a	0.945 ± 0.015 a
T300	44 ± 4 e	64.59 ± 15.74 bcd	60.96 ± 7.67 c	1.90 ± 0.18 cd	0.691 ± 0.045 de
T530	57 ± 7 cd	70.37 ± 11.39 bcd	74.66 ± 12.20 bc	1.92 ± 0.22 cd	0.660 ± 0.072 de
T1000	32 ± 5 f	37.25 ± 5.75 e	37.82 ± 7.13 d	1.58 ± 0.23 d	0.630 ± 0.093 e

Note: Various lowercase letters indicate significant differences among reclamation years (*p* < 0.05).

**Table 2:** Community diversity of bacteria in plow layer soils with different reclamation years.

Sample	OTU	Chao1	ACE	Shannon	Simpson
T0	813 ± 70 e	1144.45 ± 117.81 e	1139.84 ± 99.55 e	3.61 ± 0.27 c	0.903 ± 0.029 b
T5	4699 ± 236 c	5957.71 ± 252.12 c	5950.77 ± 290.10 c	7.22 ± 0.09 a	0.998 ± 0.000 a
T10	4475 ± 487 c	5682.08 ± 661.64 c	5735.64 ± 707.37 c	6.99 ± 0.17 a	0.997 ± 0.001 a
T30	5447 ± 324 b	6940.46 ± 413.73 b	7030.58 ± 422.81 b	7.28 ± 0.18 a	0.997 ± 0.001 a
T70	5366 ± 252 b	7018.99 ± 449.78 b	7094.17 ± 484.38 b	7.21 ± 0.24 a	0.996 ± 0.002 a
T210	6064 ± 297 a	7753.64 ± 362.18 a	7811.60 ± 346.81 a	7.33 ± 0.30 a	0.996 ± 0.003 a
T230	5667 ± 303 ab	7319.18 ± 241.53 ab	7401.93 ± 210.37 ab	7.30 ± 0.22 a	0.997 ± 0.001 a
T290	5408 ± 415 b	6920.01 ± 511.01 b	6955.94 ± 489.98 b	7.17 ± 0.28 a	0.995 ± 0.003 a
T300	4436 ± 355 c	5969.29 ± 435.63 c	6129.41 ± 410.47 c	7.06 ± 0.42 a	0.994 ± 0.006 a
T530	4428 ± 203 c	5949.33 ± 222.49 c	6129.08 ± 202.41 c	7.06 ± 0.21 a	0.996 ± 0.002 a
T1000	1743 ± 390 d	2268.92 ± 509.64 d	2261.23 ± 550.09 d	5.91 ± 0.44 b	0.986 ± 0.007 a

Note: Various lowercase letters indicate significant differences among reclamation years (*p* < 0.05).

## Data Availability

The original contributions presented in this study are included in the article/Supplementary Materials. Further inquiries can be directed to the corresponding authors.

## Supplementary Materials

The following supporting information can be downloaded at: https://www.mdpi.com/article/10.3390/microorganisms14010120/s1. Figure S1: Environmental heterogeneity in plow layer soils with different reclamation years; Figure S2: Functional heatmap of FAPROTAX predicting the functions of archaeal (A) and bacterial (B) communities in the plow layer soil under different reclamation years; Figure S3: NMDS analysis of Archaea (A) versus bacteria (B); Figure S4: βNTI value of archaeal and bacterial communities in the plow layer soil in relation to geographic distance, environmental distance, and differences in reclamation years; Table S1: Physicochemical properties in plow layer soils with different reclamation years; Table S2: Effects of different environmental factors on gene copy numbers of archaea and bacteria; Table S3: Analysis of multiple regression on similarity matrices (MRM) for prokaryotic microbial communities in plow layer soils.
