# Supplementary material for: Prokaryotic Microbial Diversity and Community Assembly in Reclaimed Coastal Agricultural Soils
Source: Microorganisms. 2026 Jan 6;14(1):120. doi: 10.3390/microorganisms14010120 (PMC12843626; doi:10.3390/microorganisms14010120)
Supplement: Supplementary file 1 [file microorganisms-14-00120-s001.zip › microorganisms-4048193-supplementary.pdf]

Supplementary Materials

Table S1. Physicochemical properties in plow layer soils with different reclamation years.

Sample	pH	EC （μs/cm）	TOC （g/kg）	RC （g/kg）	TN （g/kg）	AN （mg/kg）	AP （mg/kg）	AK （mg/kg）	CEC （cmol/kg）	PP (2-20μm/%)	SP （20μm-2mm/%）
T0	8.15±0.03a	3377.50±331.79a	4.37±0.30g	3.76±0.28def	0.56±0.08d	22.75±7.78d	3.68±1.19g	329.84±23.73a	8.98±0.43de	37.87±5.74c	62.10±5.75b
T5	8.07±0.10a	416.32±65.02bc	5.70±0.98efg	4.87±0.73cd	0.71±0.16cd	24.27±5.34d	6.98±1.23fg	130.09±26.11e	7.67±1.40e	41.96±6.85c	57.90±6.94b
T10	8.03±0.12a	265.33±63.01cd	8.32±0.76bc	7.30±0.62a	1.07±0.07ab	47.95±2.31bc	24.95±7.55de	272.75±37.70b	14.78±1.10b	42.37±3.55c	57.11±3.45b
T30	7.85±0.06ab	256.17±50.03cd	5.02±0.59efg	4.34±0.36def	0.75±0.16cd	38.97±1.84c	11.78±4.19fg	102.41±11.03e	7.11±0.37e	22.13±7.14d	77.87±7.14a
T70	8.06±0.21a	588.67±108.39b	6.59±0.64de	4.37±0.29def	0.94±0.07bc	54.48±8.00b	71.05±9.43a	225.58±41.79c	8.15±0.60e	37.82±3.86c	62.18±3.86b
T210	7.82±0.10ab	543.67±121.57b	4.82±0.28fg	3.35±0.26ef	0.86±0.19bc	40.48±4.67c	34.86±11.58d	165.34±31.59d	9.27±0.69de	41.74±1.69c	58.26±1.69b
T230	7.81±0.19ab	490.22±90.55b	5.04±0.19efg	3.27±0.16f	0.78±0.18cd	36.87±5.82c	57.25±11.66b	312.01±24.46ab	7.71±0.37e	45.05±0.72c	54.94±0.72b
T290	8.00±0.07a	133.01±16.52d	6.04±0.45def	3.74±0.33def	0.73±0.09cd	36.98±2.95c	26.87±2.39de	111.88±5.39e	8.15±0.33e	41.95±2.69c	58.04±2.70b
T300	7.93±0.17a	112.43±11.87d	8.73±2.17b	5.57±1.59bc	1.03±0.20ab	57.52±13.28b	17.94±3.50ef	108.00±10.62e	16.56±3.50a	52.76±2.34b	47.11±2.44c
T530	7.57±0.15b	229.33±48.93cd	7.34±0.80cd	4.56±0.61de	1.09±0.16ab	54.83±6.45b	75.41±9.58a	276.32±34.53b	10.87±0.32cd	53.70±1.28b	46.12±1.35c
T1000	5.76±0.37c	184.30±42.27d	10.06±0.76a	5.95±0.55b	1.22±0.20a	87.62±15.25a	45.72±11.64c	82.09±22.76e	12.16±0.53c	71.67±1.15a	28.01±1.17d

Note: Various lowercase letters indicate significant differences among reclamation years ($p < 0.05$).

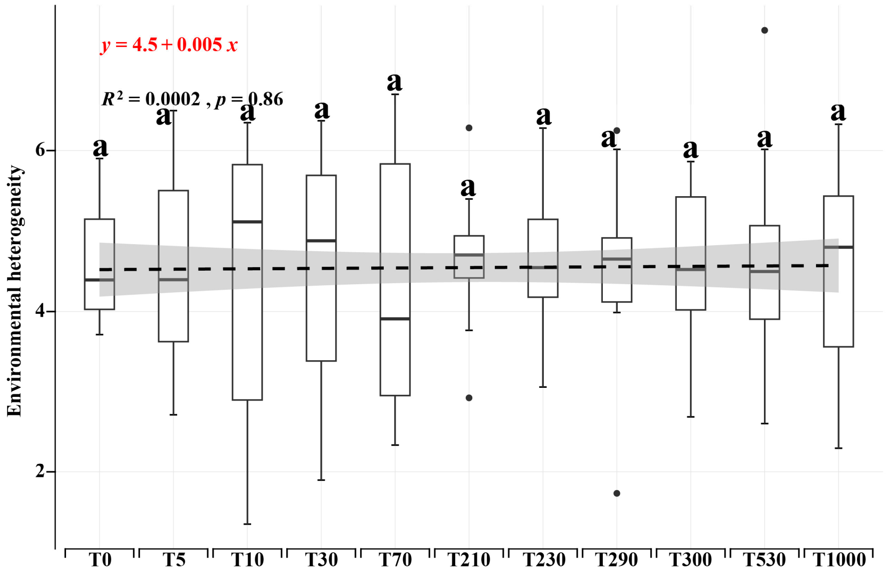

Figure S1. Environmental heterogeneity in plow layer soils with different reclamation years (Note: The dashed line represents linear fitting and lowercase letters represent difference testing)

Table S2. Effects of different environmental factors on gene copy numbers of archaea and bacteria.

	pH	EC	TOC	RC	TN	AN	AP	AK	CEC	PP	SP
Archaea 16S rRNA gene abundance	**-.40****	.04	.10	-.22	.22	**.42****	**.64****	-.07	-.19	.16	-.14
Bacterial 16S rRNA gene abundance	**.32****	**.33****	**-.43****	**-.27***	**-.51****	**-.41****	**-.33****	-.04	**-.48****	**-.42****	**.43****

Note: ** $p < 0.01$, * $p < 0.05$.

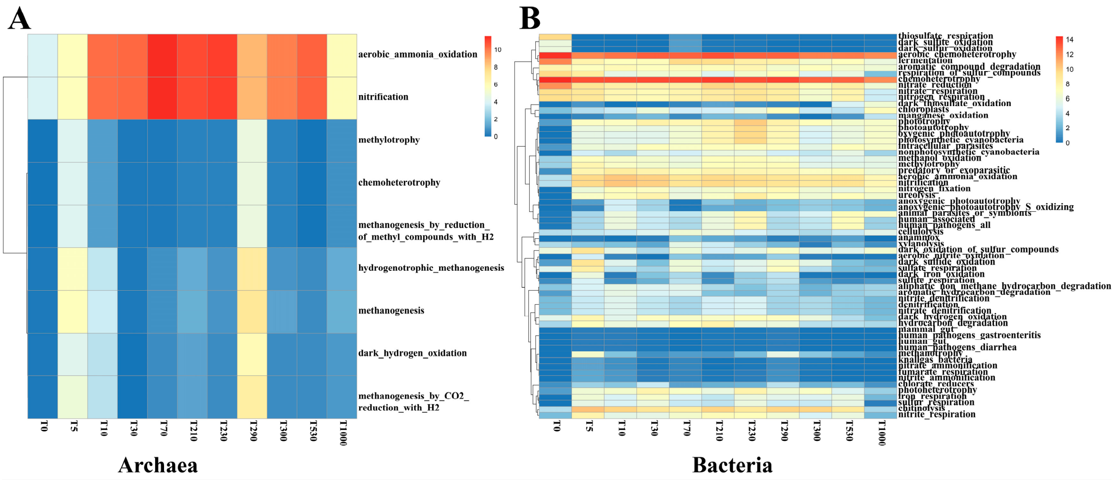

Figure S2. Functional heatmap of FAPROTAX predicting the functions of archaeal (A) and bacterial (B) communities in the plow layer soil under different reclamation years

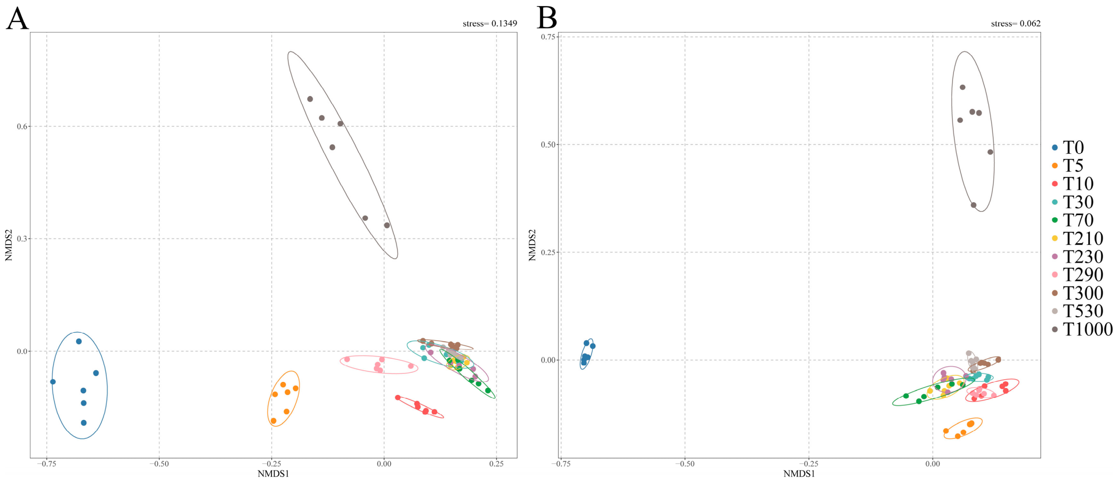

Figure S3. NMDS analysis of Archaea (A) versus bacteria (B).

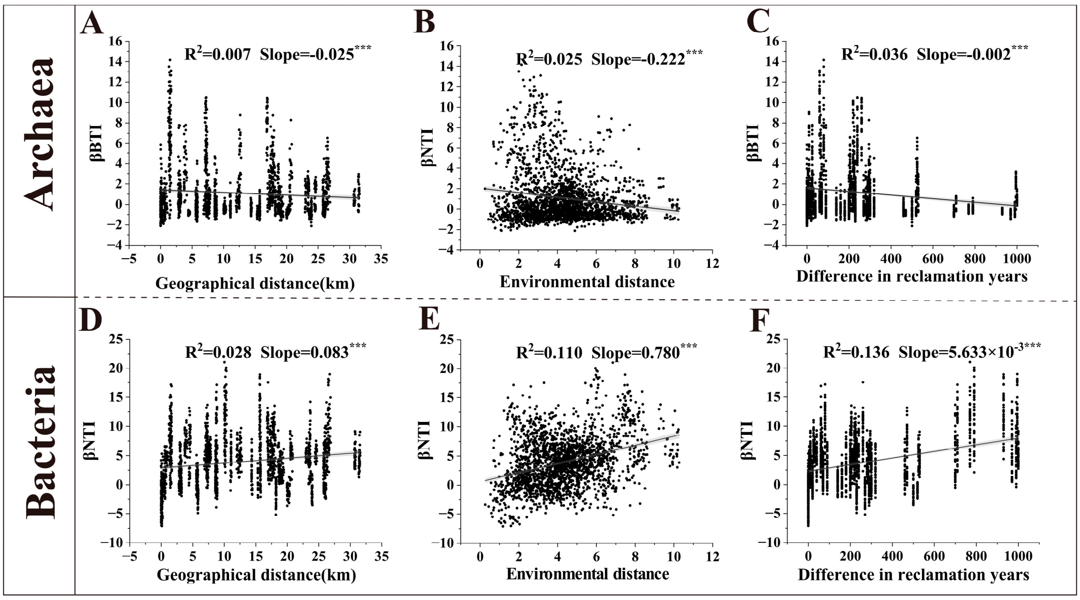

Figure S4. βNTI value of archaeal and bacterial communities in the plow layer soil in relation to geographic distance, environmental distance, and differences in reclamation years. (A, Archaeal βNTI value vs. geographic distance; B, Archaeal βNTI value vs. environmental distance; C, Archaeal βNTI value vs. difference in reclamation years; D, Bacterial βNTI value vs. geographic distance; E, Bacterial βNTI value vs. environmental distance; F, Bacterial βNTI value vs. difference in reclamation years; *** $p < 0.001$).

Table S3. Analysis of multiple regression on similarity matrices(MRM) for prokaryotic microbial communities in plow layer soils.

	archaea		bacteria	
	$R^2 = 0.496$; $p = 0.0001$***		$R^2 = 0.745$; $p = 0.0001$***	
	Coefficient	*p*	Coefficient	*p*
Geographical distance	-0.0912	0.0001***	-0.0227	0.0232*
Reclamation years	0.0741	0.0729	0.0048	0.8087
Environmental factors	-0.0544	0.8582	0.1159	0.4361
pH	-0.1223	0.0002***	-0.0744	0.0001***
EC	-0.0360	0.9062	-0.2213	0.1365
TOC	0.0696	0.0006***	0.0262	0.0079**
RC	-0.0621	0.0007***	-0.0214	0.0098**
TN	0.0185	0.1831	0.0085	0.2021
AN	-0.0782	0.0008***	-0.0312	0.0045**
AP	0.0146	0.1896	-0.0048	0.3766
AK	-0.0039	0.7017	-0.0093	0.0548
CEC	0.0006	0.9685	-0.0067	0.3878
PP	0.6273	0.1547	0.4737	0.0237*
SP	-0.6333	0.1514	-0.4851	0.0210*

Note: *** $p < 0.001$, ** $p < 0.01$, * $p < 0.05$.
